# Astrocyte Responses to Complement Peptide C3a are Highly Context-Dependent

**DOI:** 10.1007/s11064-022-03743-5

**Published:** 2022-09-12

**Authors:** Marcela Pekna, Sumen Siqin, Yolanda de Pablo, Anna Stokowska, Åsa Torinsson Naluai, Milos Pekny

**Affiliations:** 1 Laboratory of Regenerative Neuroimmunology, Department of Clinical Neuroscience, Center for Brain Repair, Institute of Neuroscience and Physiology, Sahlgrenska Academy at the University of Gothenburg, Box 440, 405 30 Göteborg, Sweden; 2 Laboratory of Astrocyte Biology and CNS Regeneration, Department of Clinical Neuroscience, Center for Brain Repair, Institute of Neuroscience and Physiology, Sahlgrenska Academy at the University of Gothenburg, Box 440, 405 30 Göteborg, Sweden; 3 Department of Laboratory Medicine, Institute of Biomedicine, Sahlgrenska Academy at the University of Gothenburg, Göteborg, Sweden; 4 Florey Institute of Neuroscience and and Mental Health, Parkville, Melbourne, Australia; 5 University of Newcastle, Newcastle, NSW Australia; 6 Present Address: Division of Episomal Persistent DNA in Cancer and Chronic Diseases, German Cancer Research Centre (DKFZ), 69120 Heidelberg, Germany

**Keywords:** Gene expression, Glia, Ischemia, Lipopolysaccharide, Neurodegeneration

## Abstract

Astrocytes perform a range of homeostatic and regulatory tasks that are critical for normal functioning of the central nervous system. In response to an injury or disease, astrocytes undergo a pronounced transformation into a reactive state that involves changes in the expression of many genes and dramatically changes astrocyte morphology and functions. This astrocyte reactivity is highly dependent on the initiating insult and pathological context. C3a is a peptide generated by the proteolytic cleavage of the third complement component. C3a has been shown to exert neuroprotective effects, stimulate neural plasticity and promote astrocyte survival but can also contribute to synapse loss, Alzheimer’s disease type neurodegeneration and blood–brain barrier dysfunction. To test the hypothesis that C3a elicits differential effects on astrocytes depending on their reactivity state, we measured the expression of *Gfap, Nes, C3ar1, C3, Ngf, Tnf* and *Il1b* in primary mouse cortical astrocytes after chemical ischemia, after exposure to lipopolysaccharide (LPS) as well as in control naïve astrocytes. We found that C3a down-regulated the expression of *Gfap, C3* and *Nes* in astrocytes after ischemia. Further, C3a increased the expression of *Tnf* and *Il1b* in naive astrocytes and the expression of *Nes* in astrocytes exposed to LPS but did not affect the expression of *C3ar1* or *Ngf.* Jointly, these results provide the first evidence that the complement peptide C3a modulates the responses of astrocytes in a highly context-dependent manner.

## Introduction

Astrocytes provide trophic support to neurons, regulate neuronal functioning and maintain brain tissue homeostasis by e.g. neurotransmitter uptake, metabolite recycling and regulation of water balance [1]. In response to infection, acute injury such as trauma or stroke, as well as chronic neurodegenerative processes, astrocytes undergo a transformation into a reactive state that is aimed at limiting tissue damage and restoration of homeostasis, and involves changes in the expression of many genes as well as alteration of astrocyte morphology and functions [2]. This astrocyte reactivity is, however, highly dependent on the initiating insult and pathological context [3] and may even be maladaptive or inhibit neuroregeneration [4–10].

Astrocytes responding to ischemia upregulate many neurotrophic genes [3] and promote neuronal survival, repair and recovery [11–16]. Systemic inflammation, typically modelled by exposure to endotoxin lipopolysaccharide (LPS), leads to the induction of reactive astrocytes that express high amounts of the third complement component (C3) [17] and lipocalin-2, and increase their secretion of long-chain saturated lipids that are toxic to neurons and oligodendrocytes [18]. In contrast to microglia, rodent astrocytes do not respond to LPS directly as they lack the required receptors and down-stream signaling components [17]. Instead, LPS exerts its effects on astrocytes via microglia-derived complement component C1q, interleukin-1a and tumor necrosis factor (TNF) [17]. Astrocytes expressing high levels of C3 are also found in aged brain [19] and in neurodegenerative diseases such as Alzheimer’s disease, Parkinson’s disease, amyotrophic lateral sclerosis, multiple sclerosis [17], or in a deafferented hippocampus [20]. In Alzheimer’s disease, C3 is also expressed by microglia and C3 secreted by both microglia and astrocytes is involved in reciprocal signaling between these glial populations to produce excess C3 [21]. Although microglia and astrocytes express complement receptors that can bind C3-derived ligands [22] that could be involved in this process, the specific receptor or mechanism for this C3-mediated reciprocal signaling between microglia and astrocytes remains to be identified.

Proteolytic cleavage of C3 by complement cascade-derived C3 convertases and other membrane-associated or serine proteases generates a larger C3b fragment and smaller C3a fragment [23]. C3b can augment complement activation and functions as opsonin in the phagocytosis of synapses and clearance of cell debris by microglia [24, 25]. Through binding to a seven transmembrane domain G-protein-coupled C3a receptor (C3aR), C3a promotes astrocyte survival [26], regulates neuronal maturation, differentiation and migration of neural stem/progenitor cells [27, 28], stimulates adult neurogenesis [29], and is neuroprotective [30–32]. In the post-ischemic brain, C3a stimulates neural plasticity and recovery [33, 34]. However, C3aR signaling has been shown to also contribute to Alzheimer’s disease type neurodegeneration [35, 36], virus-induced synapse loss and memory impairment [37], and blood–brain barrier dysfunction associated with aging [38]. Given these wide-ranging and even opposite effects of C3a-C3aR in the brain and our previous finding that the effects of C3a on neural progenitor cell migration are dependent on the concentration of stromal-derived factor [27], we hypothesized that the effects of C3a on astrocytes are context-dependent. To test this hypothesis, we cultured naïve (unchallenged) primary mouse astrocytes, astrocytes subjected to ischemia and astrocytes exposed to LPS in the presence or absence of C3a and used quantitative real time PCR (qRT-PCR) to determine the expression of genes coding for intermediate filament (nanofilament) proteins glial fibrillary acidic protein (GFAP) and nestin, C3aR, C3, nerve growth factor (NGF), tumor necrosis factor (TNF) and interleukin-1β (IL1β).

## Materials and Methods

### Primary Astrocyte Culture Preparation

Primary astrocyte cultures were prepared from postnatal day 2 C57BL/6NCr mice (Charles River) as previously described [39] with minor modifications. Briefly, mice were decapitated and brains were dissected under sterile conditions. After removal of meninges, cortices were enzymatically digested with 0.25% trypsin (Sigma-Aldrich) and DNase I (0.01 mg/ml, Sigma-Aldrich) in Hank's Balanced Salt Solution for 30 min at 37 °C. After dissociation, cells were centrifuged at 800* g* for 5 min and resuspended in astrocyte-specific medium Dulbecco’s modified Eagle’s medium (DMEM; Invitrogen, Carlsbad, California) with 10% fetal bovine serum (FBS, Life Technologies, Paisley, UK), 1% l-glutamine (Invitrogen, Carlsbad, California), and 1% penicillin–streptomycin (Invitrogen, Carlsbad, California). Cells were plated on noncoated 24- well culture plates (Sarstedt, Nümbrecht, Germany) and cultured for 7 days in a humidified CO_2_ incubator at 37 °C; medium was replaced every 3rd day until treatment. Using immunostaining with antibodies against Iba-1 as previously described [40], we determined that the cultures contained 1.76 ± 0.06% (n = 4) of microglial cells.

### Chemical Ischemia Induction and Lipopolysaccharide (LPS) Exposure

Chemical ischemia was induced as previously described [26] using 1 mM NaN_3_ and 2 mM 2-deoxy-D-glucose in saline (140.7 mM NaCl, 3 mM KCl, 1.2 mM MgSO_4_, 1 mM CaCl_2_, 2 mM NaH_2_PO_4_, 20 mM HEPES at pH 7.4) for 2 h. Thereafter, cells were allowed to recover in serum-free DMEM with B27 supplement (Gibco B27 supplement) in the presence or absence of 100 nM purified human C3a (Complement Technology, Tyler, Texas, USA) for 4 h. For LPS exposure experiments, cells were cultured in serum-free DMEM with B27 supplement containing LPS (1 ng/μL) for 6 h in the presence or absence of 100 nM purified human C3a. The same culture preparation was used for chemical ischemia and LPS exposure in a parallel experiment. In addition, an independent experiment using chemical ischemia was performed. Non-challenged naïve astrocytes were included as control; 4 replicates per treatment were used for each experiment.

### RNA Extraction and qRT-PCR

RNA extraction and qRT-PCR were performed as described previously [26]. Total RNA from each well was extracted using Qiagen RNeasy Micro Kit with DNase treatment (QIAGEN, Hilden, Germany). Reverse transcription was performed using cDNA synthesis kit (Takara Bio, Saint-Germain-en-Laye, France) using the following temperature profile: 22 °C for 5 min, 42 °C for 30 min, 85 °C for 5 min. RT-qPCR was conducted using TATAA SYBR Grand Mastermix ROX kit (TATAA Biocenter, Gothenburg, Sweden) and using the following temperature profile: 95 °C for 30 s followed by 40 cycles at 95 °C for 3 s, 60 °C for 15 s, and 72 °C for 10 s, and detected by Quant Studio Real-Time PCR System (Life Technologies). Reference genes Hprt1 (PM26FM) and Actb (PM20L) from the Mouse Endogenous Control Gene Panel (TATAA Biocenter) were used for data normalization. The following primer sequences were used: C3aR: C3ar1_fwd TGTTGGTGGCTCGCAGAT, C3ar1_rev GCAATGTCTTGGGGTTGAAA; C3: C3_fwd GCCTCTCCTCTGACCTCTGG, C3_rev AGTTCTTCGCACTGTTTCTGG; GFAP: GFAP_fwd AACCGCATCACCATTCCT, GFAP_rev CGCATCTCCACAGTCTTTACC; IL-1b: IL1b_fwd AGTTGACGGACCCCAAAAG, IL1b_rev CCACGGGAAAGACACAGG; nestin: nes_fwd GTCAGCTGAGCCTATAGTTCAACG, nes_rev AGAGTCACTCATCATTGCTGCTCC; NGF_fwd ACCACAGCCACAGACATCAA, NGF_rev GCACCCACTCTCAACAGGA; TNFa: Tnf_fwd TCCCTCCAGAAAAGACACCA, Tnf_rev CCACAAGCAGGAATGAGAA.

### Statistical Analysis

Data were analyzed by two-way analysis of variance (ANOVA) with post-hoc Sidak’s test. The assumption of Gaussian distribution was assessed using Shapiro-Wilks’s test. Data are presented as mean ± SEM after normalization to both reference genes. *P* values < 0.05 were considered as statistically significant.

## Results

### C3a Down-regulates the Expression of *Gfap* in Astrocytes After Ischemia but Does Not Affect Astrocyte Expression of *C3ar1* or *Ngf*

To determine the context-dependent effects of C3a on primary mouse astrocytes, we used astrocytes cultured in standard serum-free medium (naïve astrocytes), astrocytes after ischemic stress, and astrocytes exposed to LPS, an in vitro model of systemic inflammation or infection. The effects of LPS on astrocytes are indirect and mediated by microglia [17], therefore primary astrocyte cultures containing 1.8% microglia were used for all experiments.

**Fig. 1:**
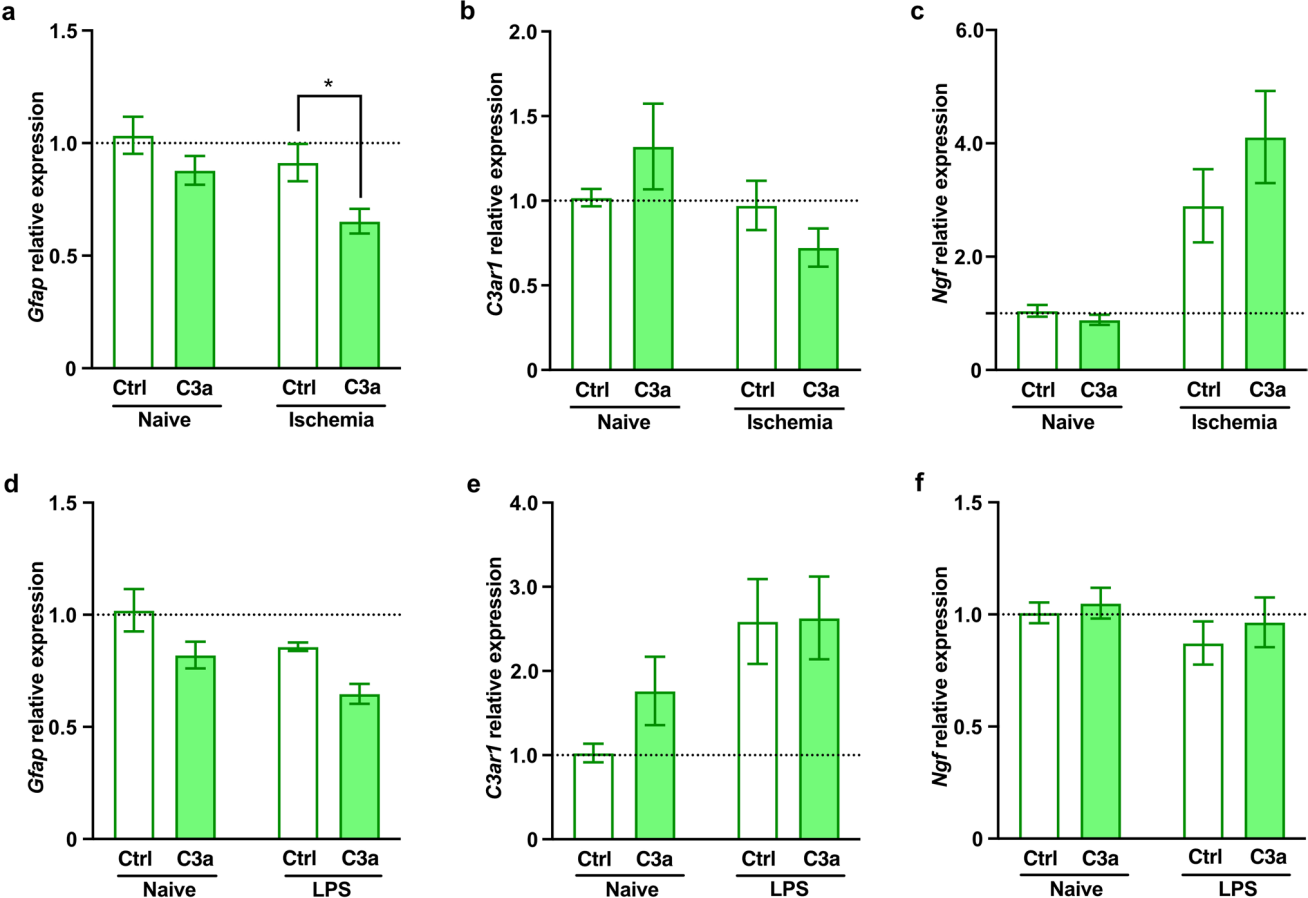
C3a down-regulates the expression of *Gfap* in astrocytes after ischemia but does not affect astrocyte expression of *C3ar1* or *Ngf.* **a** Relative expression of *Gfap,* **b** *C3ar1*, and **c** *Ngf* in naive astrocytes and astrocytes exposed to chemical ischemia for 2 h followed by 4 h recovery in the absence or presence of C3a. Values are presented as fold change compared to control cells (Ctrl). **d** Relative expression of *Gfap*, **e** *C3ar1*, and **f** *Ngf* in naive astrocytes and astrocytes after exposure to lipopolysascharide (LPS). Bar plots represent mean ± SEM. **a**–**c** *n* = 8/condition and treatment, pooled data from 2 independent experiments; **d**–**f** *n* = 4/condition and treatment. Two-way ANOVA with Sidak’s planned comparisons, *P < 0.05

As GFAP is one of the most commonly used markers of astrocyte reactivity [41], we first assessed the effects of C3a on the expression of *Gfap*. We observed that C3a reduced the expression of *Gfap* in astrocytes recovering after ischemic stress, with the same trend in naïve astrocytes and astrocytes exposed to LPS (Fig. 1a, d). Regardless of culture condition, C3a did not affect the expression of *C3ar1* (Fig. 1b, e) or *Ngf* (Fig. 1c, f).

### C3a Differentially Affects the Expression of *C3* and *Nes* in Astrocytes After Ischemic and LPS Challenge

**Fig. 2:**
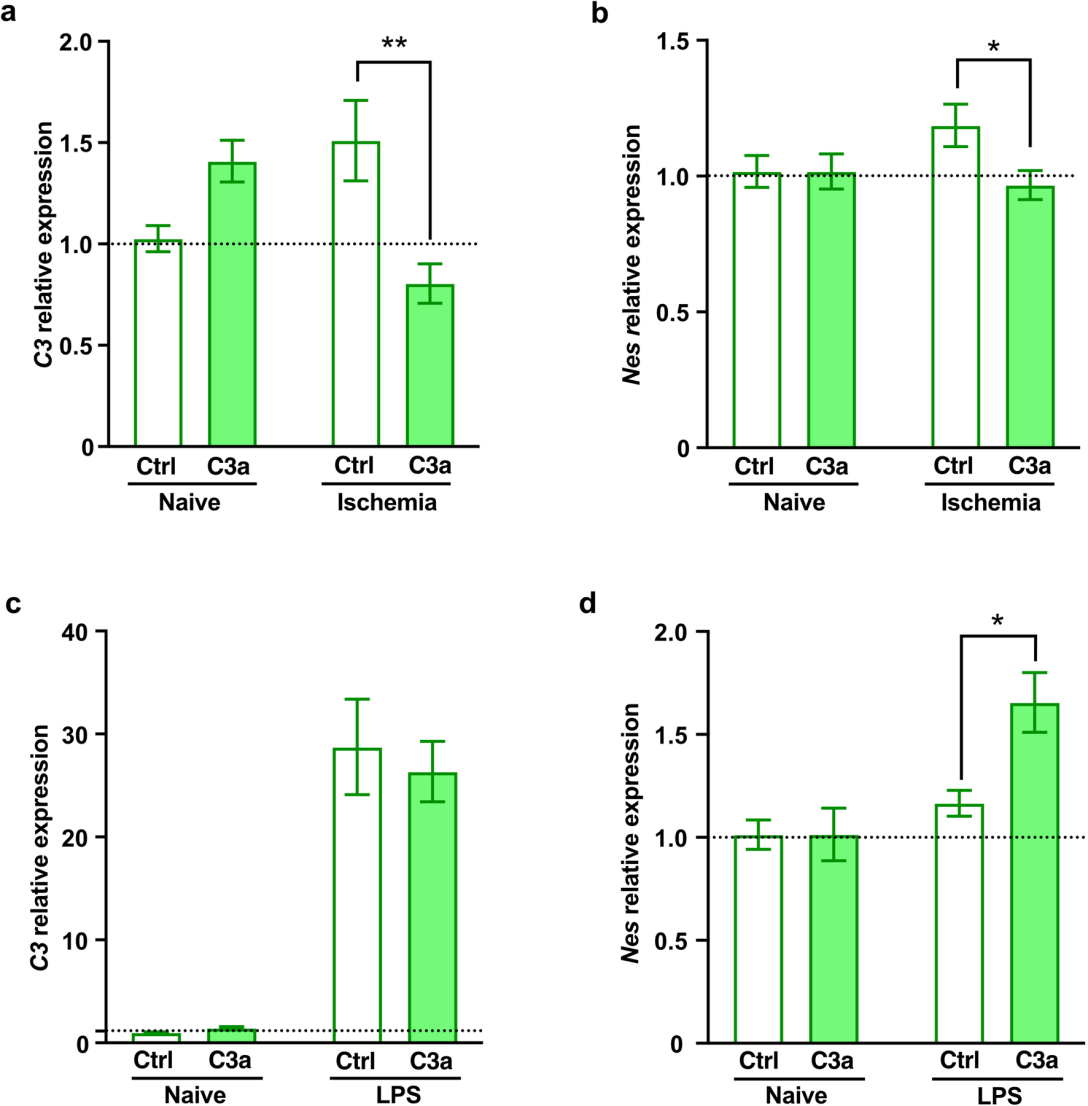
C3a differentially changes the expression of *C3* and *Nes* in astrocytes after ischemic and LPS challenge. **a** Relative expression of *C3*, and **b** *Nes* in naive astrocytes and astrocytes exposed to chemical ischemia for 2 h followed by 4 h recovery in the absence or presence of C3a. Values are presented as fold change compared to control cells (Ctrl). **c** Relative expression of *C3*, **d** *Nes* in naive astrocytes and astrocytes after exposure to lipopolysascharide (LPS). Bar plots represent mean ± SEM. **a**, **b** *n* = 8/condition and treatment, pooled data from 2 independent experiments; **c**, **d** *n* = 4/condition and treatment. Two-way ANOVA with Sidak’s planned comparisons, *P < 0.05, **P < 0.01

Next, we assessed the effects of C3a on the expression of *C3*, the product of which has been put forward as a marker of astrocytes with neurotoxic properties [17, 18]. Similar to its effect on the expression of *Gfap*, C3a down-regulated the expression of C3 in astrocytes after ischemic stress, but not in naïve astrocytes or astrocytes exposed to LPS (Fig. 2a, c). C3a reduced the expression of another reactive astrocyte marker *Nes* [42] in astrocytes after ischemic stress but increased *Nes* expression in astrocytes exposed to LPS. In naïve astrocytes, C3a had no effect on *Nes* expression (Fig. 2b, d).

### C3a Increases the Expression of *Tnf* and *Il1b* in Naive Astrocytes

**Fig. 3:**
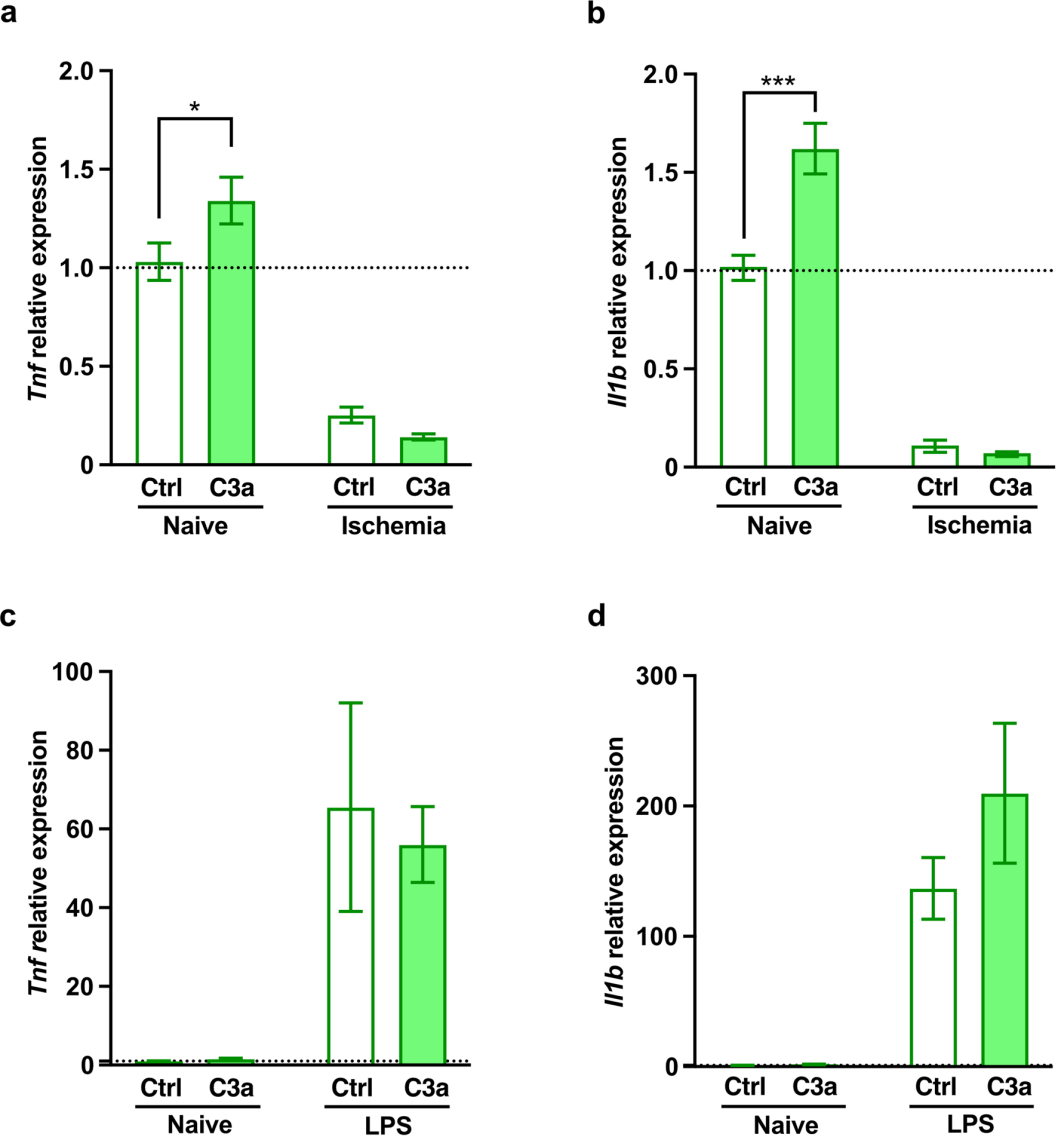
C3a increases the expression of *Tnf* and *Il1b* in naive astrocytes. **a** Relative expression of *Tnf,* and **b** *Il1b* in naive astrocytes and astrocytes exposed to chemical ischemia for 2 h followed by 4 h recovery in the absence or presence of C3a. Values are presented as fold change compared to control cells (Ctrl). **c** Relative expression of *Tnf*, **d** *Il1b* in naive astrocytes and astrocytes after exposure to lipopolysascharide (LPS). Bar plots represent mean ± SEM. **a**, **b** *n* = 8/condition and treatment, pooled data from 2 independent experiments; **c**, **d** *n* = 4/condition and treatment. Two-way ANOVA with Sidak’s planned comparisons, *P < 0.05, ***P < 0.001

In naïve astrocytes, C3a increased the expression of *Tnf* and *Il1b,* coding for pro-inflammatory cytokines TNF and IL1β (Fig. 3a, b). Exposure to LPS led to dramatic increase in the expression levels of *Tnf* and *Il1b* but the expression of *Tnf* and *Il1b* in astrocytes exposed to LPS was not affected by C3a (Fig. 3c, d).

## Discussion

Astrocyte responses to CNS insults are highly dependent on the pathological context [3]. Reactive astrocytes restore CNS homeostasis and neuronal functioning thus promoting functional recovery [11, 12, 16, 43] but reactive astrocytes may also contribute to maladaptive changes or inhibit neuroregeneration [4, 6, 44]. Here we show that the complement peptide C3a exerts differential effects on the expression of *Gfap, C3, Nes, Tnf* and *Il1b* in naïve astrocytes, astrocytes after ischemia and astrocytes exposed to LPS. These results demonstrate that C3a modulates astrocyte functions in a context-dependent manner, contribute to the understanding of the context-dependent roles of astrocytes and highlight the complexity of the effects of the complement system in the healthy and diseased CNS.

Diverse and even opposing functions for C3a-C3aR signaling in the CNS have been reported, including the regulation of neural plasticity [27, 29, 33, 34], neuroprotection [30–32], neurodegeneration [35–37], and dysfunction of blood–brain barrier [38]. The rather broad expression of C3aR on cells in the CNS, including neural progenitor cells [29] and mature neurons [45–48], microglia [49, 50], astrocytes [26, 45, 46], endothelial cells [38, 51, 52] and choroid plexus epithelium [53], could partly explain the broad range of effects of C3a in the brain. The context-dependent responses of neural progenitor cells [27] and astrocytes described here help to reconcile the seemingly conflicting findings on the effects of C3a-C3aR signaling in different types of CNS insults and pathologies. For example, C3aR deficiency protects mice against the loss of synapses in neuroinvasive viral infection [37] and C3aR antagonist treatment is protective against the reduction in synaptic density and dendritic complexity in neurodegeneration associated with Alzheimer’s disease [35] but has the opposite effects in the absence of any challenge [35], and intranasal treatment with C3a increases synaptogenesis in the peri-infarct region after focal ischemic injury [33]. Further, C3a-C3aR signaling may exert distinct effects at different stages after injury (e.g. acute versus post-acute or chronic phase after stroke), in different types of neurodegeneration (e.g. secondary neurodegeneration versus Alzheimer’s disease type neurodegeneration) or at different stages of a specific neurodegenerative condition. C3a-C3aR signaling thus evidently contributes to the nuancing of astrocyte phenotypes. Our findings provide additional argument against the binary division of reactive astrocytes into neurotoxic versus neuroprotective [41] and contribute to better understanding of the diversity of astrocyte phenotypes and functions.

We acknowledge that our study has some limitations. Due to the requirement for the presence of microglia in our astrocyte cultures to study the effects of C3a on astrocytes induced by LPS and the fact that C3aR is expressed by both astrocytes and microglia, the gene expression levels reported here reflect the combined response of both cell types. Although the relative contribution of microglia in the cultures was small, a robust microglial response could possibly mask an opposite effect of C3a on gene expression in astrocytes. In contrast to previous reports using enriched cultures of mouse and rat astrocytes [26, 54], which—similarly to ours—also typically contain 1–2% microglia [40, 55], we did not observe any increase in *Ngf* expression in astrocytes cultured in the presence of C3a. While the contribution of other cells, cell maturation stage, differences between species, as well as other differences in culture conditions could provide an explanation for this discrepancy, the effect of these factors on the net result supports the notion that the specific context plays an important role in determining astrocyte responses to C3a.

In summary, our findings show that C3a modulates astrocyte functions in a context-dependent manner and provide a potential explanation for the diverse and in some instances seemingly conflicting observations of the effects of C3a-C3aR signaling in the healthy and diseased CNS.

## Data Availability

This article contains all datasets generated or analyzed during the study.
